# The imaging of total kidney volume in ADPKD

**DOI:** 10.1590/2175-8239-JBN-2019-0218

**Published:** 2020-04-17

**Authors:** Gioacchino Li Cavoli, Francesca Finazzo, Rosalia Mongiovi, Luisa Bono, Angelo Ferrantelli, Vitalba Azzolina, Calogera Tortorici, Barbara Oliva, Antonio Amato, Carlo Giammarresi, Carmela Zagarrigo, Camillo Carollo, Franca Servillo, Onofrio Schillaci, Angelo Tralongo

**Affiliations:** 1 Azienda Ospedaliera di Rilievo Nazionale e di Alta Specializzazione Civico Di Cristina Benfratelli, Nephrology Dialysis and Renal Transplant, Palermo, Italy.; 2 Azienda Ospedaliera di Rilievo Nazionale e di Alta Specializzazione Civico Di Cristina Benfratelli, Radiology, Palermo, Italy.

**Figure1:**
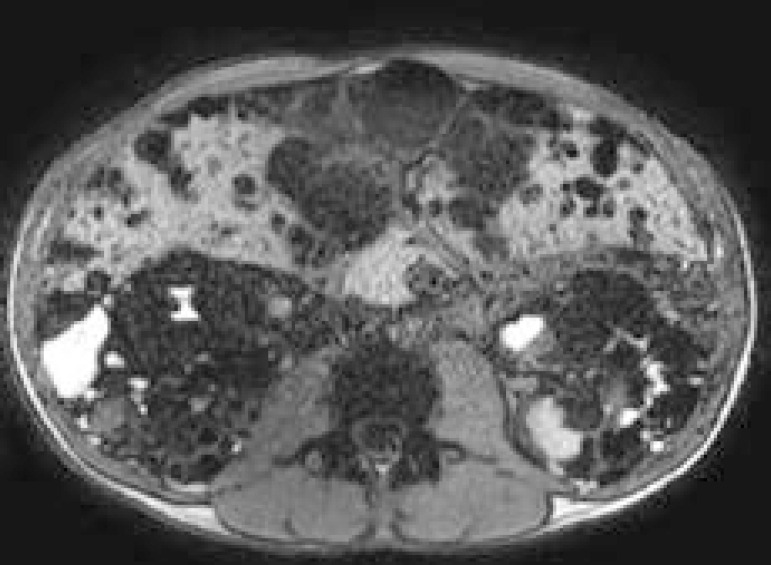
Axial T1-weighted MRI section: multiple cysts in both kidneys; most of them are hypointense (simple cysts) and some of them are hyperintense (blood breakdown products or proteinaceous or colloid contents). Cysts in liver.

Dear Editor:

Autosomal Dominant Polycystic Kidney Disease (ADPKD) is the most common inherited kidney disorder and affects up to 12 million individuals worldwide. Radiologic imaging is critical for successful management. Until recently, the treatments were only symptomatic but EMA in 2017 and FDA in 2018 approved Tolvaptan for ADPKD therapy. Renal imaging provide important diagnostic and management guidance in the monitoring of disease progression. In the past, standard radiographic imaging has not provided the accuracy necessary to reliably measure renal volume. With diagnostic advances, kidney volume growth is considered the principal surrogate marker predicting the decline of renal function in ADPKD; therefore the role of total kidney volume (TKV) has been investigated as surrogate endpoint in randomized clinical trials. TKV can be measured by ultrasonography (US), computed tomography (CT) and magnetic resonance imaging (MRI) using manual, semiautomated, or fully automated data processing techniques.^1^ US is the first step for ADPKD progression assessment but its inaccuracies limit its application in clinical practice. Regarding the monitoring of ADPKD progression, a kidney with a length of >17 cm should not be considered for US examination.^2^ Precise measurements of TKV can be obtained by planimetry or stereology analysis of CT/MRI images. CT provides accurate and reliable measurements of TKV and cyst volume in ADPKD but is not routinely used for diagnosis or follow-up studies because it exposes patients to radiation; CT is more expensive than US, which has higher availability and less cost but greater interobserver variability than MRI. This adds to the complexity of measuring disease progression over time and requires accurate recording of reformatted kidney diameters to reproduce the images. Accurate and reproducible TKV measurements, using a dose-minimizing ultra-low-dose CT protocol and volume measurement are comparable to the reference standard of MRI planimetry. Therefore ultra-low dose CT protocol represents a viable alternative where the access to MRI is limited. ERA-EDTA guidelines statement recommended that MRI be used in clinical practice for identifying ADPKD patients with rapid disease progression^3^; therefore MRI measurement of TKV is considered the gold standard for assessing the therapeutic response to new drugs developed to slow the diseases progression. (Fig. 1).

Using high-resolution MRI, the Consortium of Radiologic Imaging Studies of PKD (CRISP), observational cohort study of ADPKD subjects, investigated the ADPKD progression highlighting that MRI could accurately and reliably measure TKV cystic kidneys.^4^ The CRISP experience was later used in the Mayo Clinic Translational Polycystic Kidney Disease Center to identify image-based criteria and the height-adjusted TKV (ht-TKV) determined by CT/MRI using the ellipsoid equation, producing a practical classification in typical and atypical ADPKD patients; ht-TKV and change in eGFR over time were strongly associated in typical patients.^5^ These subjects can be assigned into one of five subclasses (1A to 1E) with increasing rate of ht-TKV accretion in time. There is growing evidence to support the importance of ht-TKV as prognostic marker in ADPKD. Patient classiﬁcation based on ht-TKV and age plays a central role in the management of these patients and allows to assess the risk of disease progression.
